# Salt-inducible kinase 1 regulates bone anabolism via the CRTC1–CREB–Id1 axis

**DOI:** 10.1038/s41419-019-1915-4

**Published:** 2019-10-31

**Authors:** Min Kyung Kim, Jun-Oh Kwon, Min-Kyoung Song, Bongjun Kim, Haemin Kim, Zang Hee Lee, Seung-Hoi Koo, Hong-Hee Kim

**Affiliations:** 1 Department of Cell and Developmental Biology, BK21 Program and DRI, School of Dentistry, Seoul National University, Seoul, 03080 Republic of Korea; 2 Arthritis and Tissue Degeneration Program, David Z. Rosensweig Genomics Research Center, Hospital for Special Surgery, 535 East 70th Street, New York, 10021 NY USA; 3 Division of Life Sciences, College of Life Sciences & Biotechnology, Korea University, Seoul, 02841 Republic of Korea

**Keywords:** Cell signalling, Osteoporosis

## Abstract

New bone anabolic agents for the effective treatment of bone metabolic diseases like osteoporosis are of high clinical demand. In the present study, we reveal the function of salt-inducible kinase 1 (SIK1) in regulating osteoblast differentiation. Gene knockdown of SIK1 but not of SIK2 or SIK3 expression in primary preosteoblasts increased osteoblast differentiation and bone matrix mineralization. SIK1 also regulated the proliferation of osteoblastic precursor cells in osteogenesis. This negative control of osteoblasts required the catalytic activity of SIK1. SIK1 phosphorylated CREB regulated transcription coactivator 1 (CRTC1), preventing CRTC1 from enhancing CREB transcriptional activity for the expression of osteogenic genes like Id1. Furthermore, SIK1 knockout (KO) mice had higher bone mass, osteoblast number, and bone formation rate versus littermate wild-type (WT) mice. Preosteoblasts from SIK1 KO mice showed more osteoblastogenic potential than did WT cells, whereas osteoclast generation among KO and WT precursors was indifferent. In addition, bone morphogenic protein 2 (BMP2) suppressed both SIK1 expression as well as SIK1 activity by protein kinase A (PKA)–dependent mechanisms to stimulate osteogenesis. Taken together, our results indicate that SIK1 is a key negative regulator of preosteoblast proliferation and osteoblast differentiation and that the repression of SIK1 is crucial for BMP2 signaling for osteogenesis. Therefore, we propose SIK1 to be a useful therapeutic target for the development of bone anabolic strategies.

## Introduction

An imbalance in the amount of bone-formation by osteoblasts and bone resorption by osteoclasts in bone remodeling leads to bone metabolic diseases like osteoporosis. For several decades, osteoclast-targeted anti-resorptive drugs have been developed for the treatment of osteolytic disorders^1^. More recently, osteoblast-targeted anabolic agents were developed for osteoporosis and fracture treatments^2,3^. However, these agents have revealed several side effects, placing limitations to clinical usage^3–5^. Therefore, discovery of new therapeutic targets to increase anabolic activity without evoking unwanted effects is required.

Osteogenesis is a multistep process involving osteoblast differentiation, production of organic bone matrix (osteoid), and mineralization of osteoid. Osteoblast differentiation is achieved through a series of ill-demarcated steps including the commitment of mesenchymal stem cells to osteoprogenitors, proliferation of progenitor cells, early differentiation to immature osteoblasts, and differentiation to mature osteoblasts^6,7^. These multiple steps are regulated by diverse groups of extracellular signals like bone morphogenic protein (BMP), Wnt, hedgehog, and parathyroid hormone-related peptide (PTHrP) via distinct or overlapping intracellular signaling pathways^6^.

BMPs elicit osteogenic responses by binding to heteromeric receptor complexes. The activated receptor phosphorylates and thereby activates Smad1/5/9, which forms a complex with Smad4 and subsequently translocate the nucleus. Smad stimulates Runx2 expression and cooperates with Runx2 for induction of other transcription factors like the inhibitors of DNA binding/differentiation (Ids), Osterix (OSX), and Dlx^8,9^. Besides this Smad-dependent pathway, non-Smad pathways contribute to BMP signaling in osteogenesis. The most well-described non-Smad pathway is the TAK1–MKK–MAPK axis that facilitates the activation and also expression of Runx2^10–12^. As a result, BMP signaling induces expression of genes needed for osteoblast function like alkaline phosphatase (ALP), collagen type I alpha 1 (COL1A1), and osteocalcin (OCN)^13^.

The salt-inducible kinase (SIK) family belongs to the AMPK family and contains three members: SIK1, SIK2, and SIK3^14^. Although SIKs share structural domains, SIK1 is different from SIK2 and SIK3 in that SIK1 expression is regulated by external stimuli in contrast to the constitutive expression of SIK2 and SIK3^15^. The activity of SIKs is stimulated by phosphorylating in the N-terminal kinase domain by LKB^16,17^. In contrast, phosphorylation in the C-terminal domain by PKA inhibits SIKs activity^18,19^. The best-described downstream targets of SIKs are CREB regulated transcription coactivator (CRTC; also called TORC) proteins^20–22^ and class IIa histone deacetylases (HDACs)^23,24^. Recently, TAB2 was shown to be a target of SIK1 and SIK3 in Toll-like receptor signaling^25–27^. The phosphorylation of CRTCs by SIKs leads to cytoplasmic sequestration of CRTCs, preventing the nuclear action of CRTCs in potentiating CREB activity as a coactivator. Therefore, SIK negatively regulates CREB-mediated responses, while PKA can negate SIK-mediated CREB inhibition^22,28^.

The elevation of cAMP levels or PKA activity has been implicated in bone anabolism. In addition, protein kinase A (PKA) activity was shown to regulate BMP signaling^29–31^. In these responses, PKA has been thought to directly activate CREB by phosphorylating it^32^. CREB binds CRE on the promoters of osteogenic genes such as Id1, bone sialoprotein (BSP), and osteocalcin (OCN), stimulating their transcription^33–35^. Although the bone anabolic role of PKA has been mostly described for GPCR-stimulating signals like PTH^32,36^, several studies have shown that PKA activation is also induced by BMPs during osteogenesis^29–31^.

The regulation of SIKs by PKA and the bone anabolic role of the PKA pathway may imply the presence of a crosstalk between these kinases in skeletal development and metabolism. Indeed, SIK3 knockout (KO) mice showed a severe inhibition of chondrocyte^37,38^. In addition, SIK2 and SIK3, highly expressed in osteocytes, were shown to exert a negative regulatory function in PTH-stimulated bone formation by suppressing the expression of sclerostin, a Wnt inhibitor^37,38^. However, the role and molecular mechanism of SIK1 in the skeletal system has not been investigated. In this study, we found that SIK1 regulates bone metabolism by modulating osteogenesis without affecting osteoclasts.

## Materials and methods

### Animals

SIK1 KO mice (C57BL/6) were previously described^39^. All experimental procedures were approved by the Institutional Animal Care and Use Committee of Seoul National University. Mice were housed in the SPF animal facility with a 12-h day–night cycle. Neonatal ICR mice for the isolation of osteoblast precursors (preosteoblasts) and the experiments of ex vivo bone cultures were purchased from OrientBio (Seongnam, Korea).

### Reagents

Recombinant human BMP2, M-CSF, and RANKL were purchased from PeproTech (Rocky Hill, NJ, USA). Phospho-specific (Ser151) and pan-CRTC1 antibodies were purchased from Cell Signaling Technology (Danvers, MA, USA). HG-9-91-01 was purchased from MedChemExpress (Princeton, NJ, USA). Antibodies against SIK1, Id1, Lamin B, and α-tubulin, were obtained from Santa Cruz Biotechnology (Santa Cruz, CA, USA). OCN antibody was acquired from Abcam (Cambridge, UK). Anti-β-actin and anti-HA were acquired from Sigma-Aldrich (St. Louis, MO, USA). HRP Anti-Rabbit IgG (Peroxidase) Polymer Detection Kit was purchase from Vector Laboratories (Burlingame, CA, USA). Additionally, an alkaline phosphatase (ALP) assay kit was purchased from Takara Bio Inc. (Ohtsu, Japan), while an ALP staining kit, tartrate-resistant acid phosphatase (TRAP) staining kit, β-glycerophosphate, and ascorbic acid were purchased from Sigma-Aldrich (St. Louis, MO, USA). Mouse RANKL and OPG ELISA kits were purchased from R&D Systems (Minneapolis, MN, USA). pcDNA3-SIK1-HA, pcDNA3-SIK1-FLAG, and pcDNA3-SIK1-T182A-FLAG plasmids were as previously described^39^. pGL-Id1 (−1231/+88) plasmid was provided by Dr. Korchynskyi (National Academy of Sciences in Ukraine)^40^. siRNA oligonucleotides for control, SIK1, SIK2, SIK3, or CRTC1 were purchased from Thermo Fisher Scientific (Waltham, MA, USA).

### Osteoblast differentiation

Primary preosteoblasts were prepared from calvarae of neonatal mice, as described previously^41,42^. For the induction of osteogenesis, preosteoblasts were seeded at 3.5 × 10^4^ per well in 48-well plates or 3.5 × 10^5^ per well in 6-well plates in α-MEM containing 10% FBS. On the next day, the medium was changed to an osteogenic medium containing 10 mM of β-glycerophosphate (β-GP) and 100 μg/mL of ascorbic acid (AA) in α-MEM/10% FBS. Alternatively, cells were cultured with recombinant hBMP2 (150 ng/mL), a well-characerized osteogenic factor^43–45^, in α-MEM/10% FBS. For osteoblastic differentiation of the C2C12 myoblastic cell line, cells were seeded at 0.7 × 10^4^ per well in 48-well plates or 0.7 × 10^5^ per well in 6-well plates in DMEM/10% FBS and the medium was changed on the next day to the one containing hBMP2 (150 ng/mL). To qualitatively evaluate the differentiation extent, cells were stained for ALP activity. Alternatively, cells were lysed and cell lysates were subjected to quantitative ALP activity assay. Matrix mineralization was assessed by staining the cultures with Alizarin Red S. In quantitative experiments, the stained cells were incubated with 100 mM of cetylpyridium chloride for 2 h at 37 °C and the extracts were subsequently subjected to spectrophotometry at 415 nm.

### In vitro gene knockdown

Preosteoblasts were seeded at 3.5 × 10^4^ per well in 48-well plates or 3.5 × 10^5^ per well in 6-well plates and C2C12 were seeded at 0.7 × 10^4^ per well in 48-well plates or 0.7 × 10^5^ per well in 6-well plates. After culturing overnight, siRNA oligonucleotides (40 nM) were transfected into cells using HiPerFect (Qiagen, Venlo, Netherlands) following the manufacturer’s protocol. In double knockdown experiments, the concentration of each siRNA was 25 nM. On the following day, the cells received the osteogenic medium or BMP2 for differentiation. The expression level of protein or mRNA was determined at two days after transfection. The sequences of customized oligonucleotides are shown in Supplemental Table 1.

### In vitro gene overexpression

Preosteoblasts were seeded at 5 × 10^4^ per well in 48-well plates or 5 × 10^5^ per well in 6-well plates, and C2C12 were seeded at 1 × 10^4^ per well in 48-well plates or 1 × 10^5^ per well in 6-well plates. After culturing overnight, cells were transfected with pcDNA3 (EV), pcDNA3-SIK1-WT, or pcDNA3-SIK1-T182A constructs utilizing PolyFect (Qiagen, Venlo, Netherlands) according to the manufacturer’s instructions. On the next day, the cells were treated with the osteogenic medium or BMP2 for differentiation.

### Luciferase reporter assay

To assay the activity of gene promoter, C2C12 cells were seeded at 1 × 10^4^ per well in 48-well plates. Cells were transfected on the next day with CRE-Luc or Id1-Luc reporter construct using PolyFect (Qiagen, Venlo, Netherlands) according to the manufacturer’s instructions. After incubation for 12 h, cells were stimulated with hBMP2 (150 ng/mL) for 12 h. The cells were lysed with the Glo lysis buffer (Promega Corp., Madison, WI, USA) and cell lysates were subjected to luciferase assay with the Bright-Glo luciferase assay kit and GloMax96 luminometer (Promega Corp., Madison, WI, USA). Luciferase activity was normalized to the protein concentration of each sample.

### Cell proliferation assay

Calvarial preosteoblasts (1 × 10^4^) or C2C12 (0.25 × 10^4^) cells were seeded onto 96-well plates. From the next day (day 0) cell were cultured in osteogenic conditions with the medium changed every day. Cell proliferation assay was performed daily with either the BrdU cell proliferation assay kit (Millipore Corp., MA USA) or the CCK-8 kit (Dogindo Molecular Technology, Japan), following the manufacturers’ protocols.

### Osteoclast differentiation

Bone marrow–derived monocyte/macrophages (BMMs) were prepared, as previously described^46^. In brief, bone marrow cells were isolated from the tibiae and femurs of female five-week-old littermate WT or SIK1 KO mice and cultured in α-MEM containing 10% FBS overnight on culture dishes. Nonadherent cells were collected and cultured in the presence of M-CSF (30 ng/mL) for three days on Petri dishes. Attached cells (BMMs) were harvested and seeded at 3.5 × 10^4^ per well in 48-well plates or 3.5 × 10^5^ per well in 6-well plates with M-CSF (30 ng/mL). From the following day, cells were cultured with M-CSF (30 ng/mL) and RANKL (150 ng/mL) for four days to induce osteoclast differentiation. TRAP staining was performed in accordance with the manufacturer’s instructions.

Real-time PCR analysis Analyses of mRNA expression levels by real-time PCR were carried out as previously described^46^. The primers used for real-time PCR analyses are listed in Supplemental Table 2.

### Western blotting

Cells were lysed with RIPA buffer (50 mM Tris-HCl, pH: 8.0, 150 mM NaCl, 0.1% SDS, 1% NP40, 0.5% sodium deoxycholate, 0.5 mM PMSF, proteinase inhibitor cocktail, 1 mM Na_3_VO_4_, and 1 mM NaF). Following protein quantification with the DC Protein Assay Kit (Bio-Rad Laboratories, Hercules, CA, USA), equal amounts of lysates were subjected to Western blotting as described previously^47^.

### Extraction of cytoplasmic and nuclear proteins

Cytoplasmic and nuclear fraction proteins were prepared using the NE-PER Nuclear and Cytoplasmic Extraction Kit (Thermo Fisher Scientific, Waltham, MA, USA) according to the manufacturer’s instructions. In brief, cells were washed with cold PBS twice and treated with the cytoplasmic lysis buffer. After centrifugation, the supernatant was transferred to a new tube as cytoplasmic protein and the pellet was washed and treated with the nuclear lysis buffer. After centrifugation, nuclear protein was obtained from the supernatant.

### Immunocytochemistry

To detect the protein expression of SIK1 by confocal microscopy, cells cultured on cover glasses were fixed with 3.7% formaldehyde and permeabilized with 0.1% Triton X-100. After blocking with PBS containing 1% BSA, cells were incubated overnight with anti-SIK1 antibody at 4 °C. Prior to incubation with Cy3-conjugated secondary antibody. Cover glasses were mounted with a mounting medium containing DAPI (Vector Laboratories, Burlingame, CA, USA) and observed under a confocal microscope (LSM700; Carl Zeiss AG, Oberkochen, Germany).

### Calvarial organ culture and immunohistochemistry

Calvarial organ cultures were performed as previously described with slight modifications^48^. In brief, calvariae from four-day-old ICR mice (*n* = 9 per group) were cultured in IVF organ culture dishes (Corning Inc., Corning, NY, USA) in BGJB medium (Thermo Fisher Scientific, Waltham, MA, USA). The organs were transfected with the control or SIK1 siRNA (600 nM) and cultured for seven days with BMP2 (200 ng/mL). The medium was changed every two days. For analyzing mRNA, calvariae were grounded after freezing and total RNA was isolated with Trizol (Thermo Fisher Scientific, Waltham, MA, USA). For immunohistochemistry, calvariae were fixed in 4% paraformaldehyde for overnight and decalcified with 12% EDTA (pH 7.4) for 14 days before embedding in paraffin. Coronal sections prepared in 5-μm thickness were deparaffinized in xylene and rehydrated in ethanol. The sections were heated in 10 mM sodium citrate (pH 6.0) for 20 min for antigen retrieval. To block endogenous peroxidase activity, the sections were incubated for 20 min in 3% H_2_O_2_ in methanol. Then, the sections were incubated overnight with anti-OCN at 4 °C. Following incubation with a HRP polymer-conjugated secondary antibody for 30 min, horseradish peroxidase activity was visualized using the DAB Peroxidase Substrate Kit (Vector Laboratories, Burlingame, CA, USA). The sections were counterstained with Gill’s hematoxylin (Vector Laboratories). The stained images were captured with a camera-equipped microscope and OCN-positive cells on bone surfaces around the sagittal suture were scored.

### Calcein double labeling

Female WT or SIK1 KO mice (eight weeks old; *n* = 9 per group) were injected intraperitoneally with 20 mg/kg of calcein (Sigma-Aldrich, St. Louis, MO, USA) dissolved in 2% sodium bicarbonate at one and seven days before sacrifice on day 10. Femurs were fixed in 4% paraformaldehyde and undecalcified bones were embedded in methyl methacrylate. Sliced sections (5 μm) were observed under a confocal microscope (LSM700; Carl Zeiss AG, Oberkochen, Germany) and the distance between the calcein deposited bands was measured.

### μCT analysis and histomorphometry

Femurs of 10-week-old littermate WT and SIK1 homozygous KO female mice (*n* = 9 per group) and male mice (*n* = 5 per group) were analyzed using the SkyScan 1172 μCT scanner (70 kV, 141 μA, 6.92 pixel size; Skyscan, Aartselaar, Belgium). Trabecular bones were analyzed in 1-mm thickness area of distal femurs, starting from 1-mm below the growth plate and cortical bones were measured in the 1-mm thickness region starting from 3.5-mm below the growth plate where trabeculae were absent the thresholds were minimum 75 and maximum 255. Three-dimensional images were reconstituted using CT-volume software (Skyscan). For histological study, the fixed femurs were decalcified and embedded in paraffin. Serial sections (5 μm) of paraffin blocks were subjected to Goldner’s trichrome and TRAP staining. The images were captured by bright field microscopy and histomorphometric analysis was carried out with the Osteomeasure software program (Osteometrics, Inc., Decatur, CA, USA) as described previously^49^. In the sections stained with Goldner’s trichrome, osteoblast parameters were analyzed by measuring the values of area and surface perimeter of trabecular bones and the number of osteoblastic cells lining trabecular bone surface in a region below the growth plate with the same region of interest (ROI). To determine osteoclast parameters, the values of area and surface perimeters of trabecular bones and the number of TRAP-positive cells on bone surfaces were measured in a region below the growth plate with the same ROI.

### Statistical analysis

All quantitative data are shown as the means ± SDs. All in vitro experiments were repeated three to five times. The ex vivo calvaria culture experiment was repeated twice and the in vivo analysis of femurs was performed once for each sex. The significance of differences between two groups was determined using a Student’s *t*-test. The comparison of multiple groups was carried out by using a one-way analysis of variance. A *P* value of less than 0.05 was considered to be significant.

## Results

### SIK1 deficiency enhances osteogenesis in vitro and ex vivo

**Fig. 1:**
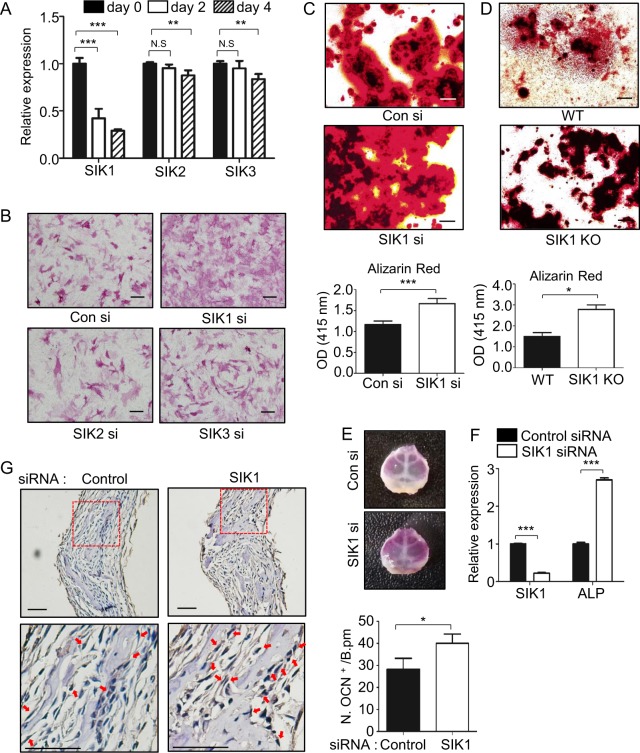
SIK1 knockdown enhances osteogenesis in vitro and ex vivo. **a** Primary preosteoblasts were treated with osteogenic medium containing β-GP and AA. The mRNA levels of SIK members were analyzed by real-time PCR. **b**–**c** siRNA-transfected cells were cultured in osteogenic medium. Cells were stained for ALP activity at day 3 *(B)*. Matrix mineralization was evaluated by Alizarin Red staining at day 12, followed by stain extraction and spectrophotometry *(C)*. **d** Primary preosteoblasts isolated from SIK1 KO or WT mice were cultured with osteogenic medium. Differentiated cells were subjected to Alizarin Red staining at day 14. **e**–**g** Calvariae from four-day-old mice were transfected with control siRNA or SIK1 siRNA and cultured with hBMP2 (200 ng/mL) for seven days ex vivo. Calvariae were stained for ALP (*E*) or analyzed for mRNA levels of SIK1 and ALP by real-time PCR *(F)*. Calvarial sections were immunostained with anti-OCN. OCN-positive cells on bone surface around the sagittal suture were scored. Magnified images of the red boxes in upper panels are shown in bottom panels with arrows indicating OCN-positive cells *(G)*. Scale bars, 200 μm in *B* and *E* and 50 μm in *G*. ***p < 0.001; **p < 0.01; *p < 0.05. *t*-test

To determine relevance of SIKs to the regulation of osteogenesis, we first examined whether the expression levels of SIKs change during the osteoblast differentiation. In osteogenic culture with medium containing β-glycerophosphate and ascorbic acid of primary mouse precursor cells, the SIK1 mRNA levels were dramatically decreased within two days, whereas the mRNA levels of SIK2 and SIK3 were almost constant until the late stage (Fig. 1a). The protein level of SIK1 was also prominently reduced in osteogenic medium (Supplemental Fig. 1). Next, we downregulated the level of each SIK with siRNA in preosteoblasts to evaluate the potential function of SIKs in osteogenesis. Specific gene knockdown was achieved for each SIK (Supplemental Fig. 2A). In SIK1 knockdown cells, we observed elevated levels of staining and quantitative activity of ALP, an osteoblast differentiation marker (Fig. 1b and Supplemental Fig. 2B, C). In contrast, SIK2 or SIK3 knockdown had little effect on ALP staining under the conditions in which the extents of knockdown efficiency were similar (Fig. 1b and Supplemental Fig. 2A), suggesting a specific role of SIK1 in controlling osteoblast differentiation. The mRNA levels of osteogenic genes OSX, ALP, and COL1A1 were significantly increased by SIK1 siRNA (Supplemental Fig. 2D). The SIK1 deficiency also enhanced matrix mineralization activity, as revealed by Alizarin Red staining (Fig. 1c). Consistently, SIK1 knockdown accelerated the BMP2-induced osteoblastic differentiation of C2C12 cells (Supplemental Fig. 3). In addition, we utilized preosteoblasts from WT or SIK1 KO mice for in vitro differentiation. Gene KO of SIK1 did not affect the SIK2 and SIK3 expression levels (Supplemental Fig. 4A). The ALP and Alizarin Red staining showed greater differentiation and mineralization activity in the SIK1 KO than WT (Fig. 1d and Supplemental Fig. 4B). In line with these staining results, the expressions of osteogenic genes were significantly elevated in SIK1 KO (Supplemental Fig. 4C).

To gain further evidence for the function of SIK1 in osteogenesis, we next evaluated the effect of SIK1 knockdown ex vivo. Calvarial bones of neonatal mice were cultured in the presence of control siRNA or SIK1 siRNA together with BMP2. With the verification of downregulation of SIK1, elevation in ALP staining and mRNA level was observed in SIK1 knockdown calvariae (Fig. 1e, f). In histological analyses, the number of OCN^+^ osteoblasts was increased by SIK1 siRNA (Fig. 1g). Collectively, these results suggest that SIK1 negatively regulates osteoblast differentiation and matrix mineralization in vitro and ex vivo.

### SIK1 regulates osteogenesis by affecting preosteoblast proliferation

**Fig. 2:**
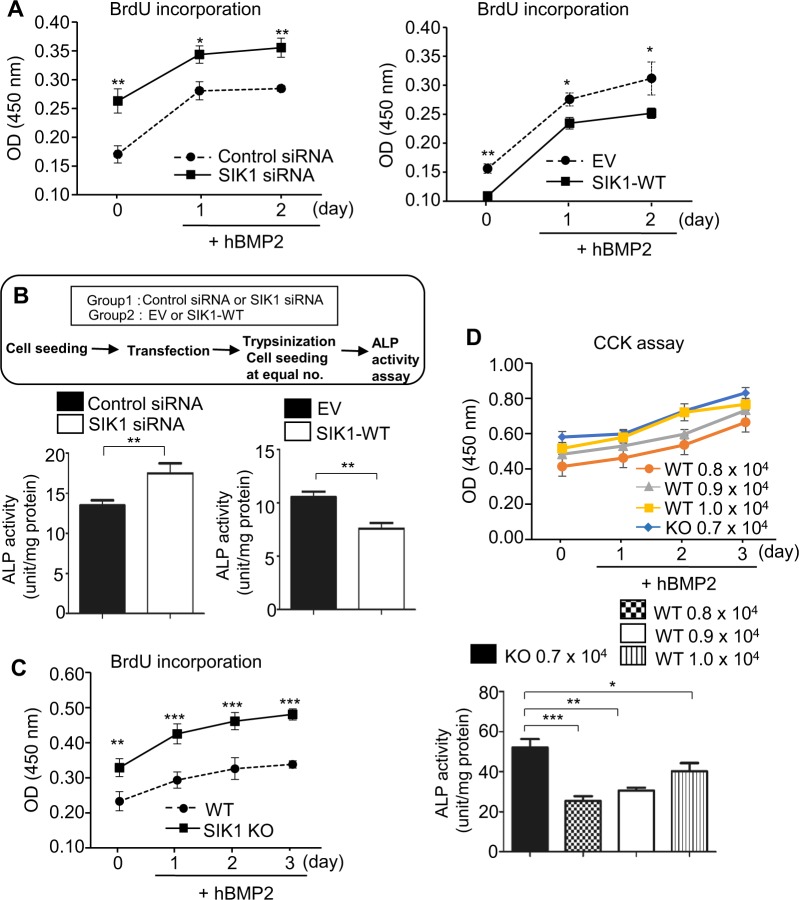
SIK1 regulates osteogenesis by affecting both proliferation and differentiation. **a** C2C12 cells were transfected with control or SIK1 siRNA (left), or with control EV or SIK1-WT expression plasmids (right). Cells were cultured with hBMP2 (150 ng/ml) for the indicated days and subjected to the BrdU incorporation assay. **b** After transfection of C2C12 cells with siRNA or expression plasmids and overnight cultue, cells were reseeded at the same density and treated with BMP2 for 2 days. ALP activity was measured with cell lysates. **c** Calvarial preosteoblasts from WT and SIK1 KO mice were treated with hBMP2 (150 ng/ml) for 3 days and subjected to the BrdU incorporation assay. **d** Calvarial preosteoblasts from WT and SIK1 KO mice were seeded at the indicated density and treated with BMP2 (150 ng/ml) for 3 days. The CCK assay and ALP activity assay were performed. ***p < 0.001; **p < 0.01; *p < 0.05. *t*-test

In osteoblastic differentiation cultures of calvarial preosteoblasts and C2C12 cells, we observed a tendency of a decrease in cell number by SIK1 deficiency (Fig. 1b and Supplemental Figs. 3 and 4B). In quantitative assays, SIK1 knockdown increased, while SIK1 overexpression decreased, the proliferation of C2C12 cells (Fig. 2a). To override the effect of different cell number at the start of differentiation culture, we re-seeded equal number of C2C12 cells that had been transfected with siRNA or overexpression plasmid at the initiation of differentiation induction. In these cultures, ALP activity was still higher in SIK1-knockdowned cells and lower in SIK1 overexpressing cells (Fig. 2b). Similarly, SIK1 WT preosteoblasts showed lower proliferation compared with SIK1 KO cells (Fig. 2c). Even when the seeding density of WT cells was modulated to have the number of cells similar to that of KO cells during the culture, ALP activity was significantly lower in WT than in KO cell culture (Fig. 2d). These data suggest that SIK1 regulates osteogenesis by modulating both the proliferation of precursor cells and the expression of osteoblastic genes.

### Kinase activity is required for SIK1 regulation of osteogenesis

**Fig. 3:**
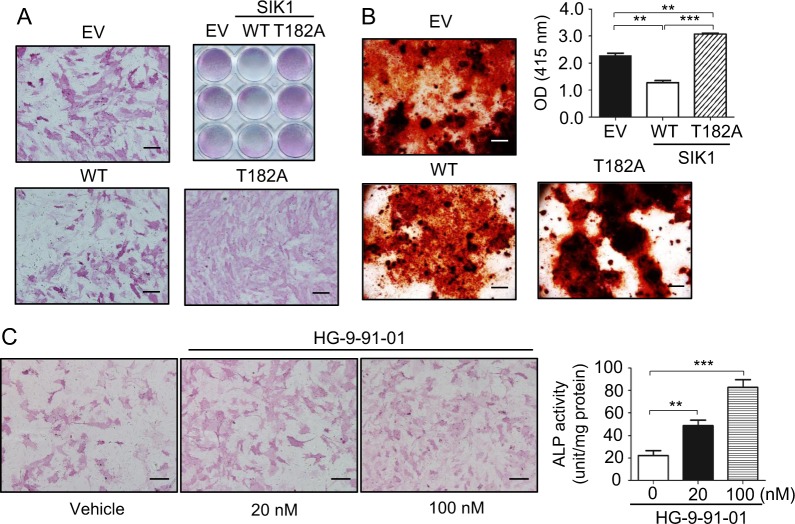
The kinase activity of SIK1 is important for osteogenesis regulation. **a** Primary preosteoblasts transfected with SIK1-WT, SIK1-T182A, or the control plasmid (EV) were cultured in osteogenic medium for four days. Cells were stained for ALP. **b** Primary preosteoblasts transfected as in *B* were cultured in osteogenic medium for 14 days before Alizarin Red staining. **c** Primary preosteoblasts were cultured in osteogenic medium containing either vehicle (DMSO) or HG-9-91-01 for three days. Cells were then stained for ALP (left) or subjected to quantitative ALP activity assay (right). ***p < 0.001; **p < 0.01. *t*-test. Scale bars, 200 μm

In contrast to the positive effect of SIK1 downregulation on osteogenesis, the overexpression of SIK1-WT decreased ALP staining and expression of osteoblastic genes in primary preosteoblasts and C2C12 cells (Fig. 3a and Supplemental Figs. 5 and 6). The Alizarin red staining was also much weaker in SIK1-overexpressing than in control cells (Fig. 3b). As SIK1 is a kinase that affects gene expression by phosphorylating transcription regulators, we next investigated whether SIK1 modulates osteogenesis by its kinase activity by employing a kinase-dead form of SIK1 (T182A)^16^. SIK1-T182A overexpression increased ALP staining and matrix mineralization activity compared with the control EV cells (Fig. 3a, b and Supplemental Fig. 7). We next examined the effect of HG-9-91-01, a pan SIK kinase inhibitor, on osteoblast differentiation. HG-9-91-01 treatment increased ALP staining and activity at concentrations as low as 20 nM in the osteogenic culture (Fig. 3c). These data indicate that the kinase activity of SIK is important to its inhibitory role in osteoblast differentiation and matrix mineralization.

### CRTC1 mediates osteogenesis regulation by SIK1

**Fig. 4:**
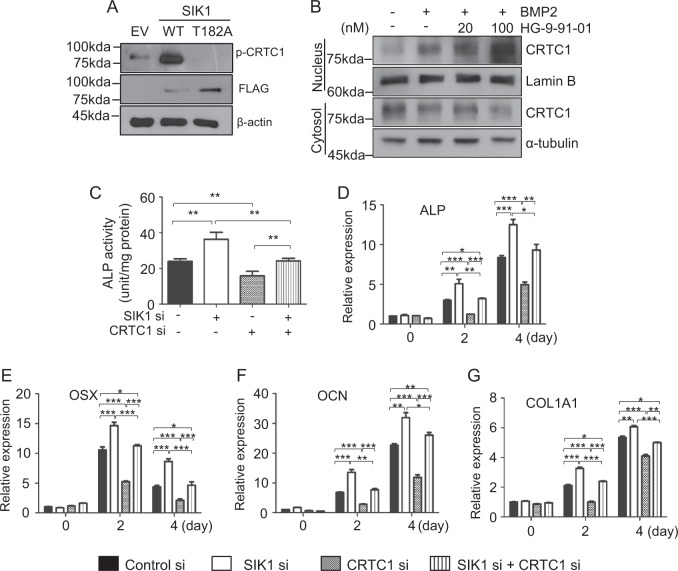
SIK1 modulates osteogenesis though CRTC1. **a** SIK1-WT-FLAG, SIK1-T182A-FLAG, or the control plasmid (EV) were transfected to C2C12 cells. After stimulation with hBMP2 (500 ng/mL) for 15 min, cell lysates were prepared and subjected to Western blotting to assess CRTC1 phosphorylation. **b** Primary preosteoblasts were stimulated with hBMP2 in the presence of HG-9-91-01 for 20 min. Nuclear and cytoplasmic CRTC1 proteins were analyzed by Western blotting. Lamin B and α-tubulin were used as loading controls for nuclear and cytoplasmic extracts. **c**–**h** Primary preosteoblasts were co-transfected with SIK1 siRNA and CRTC1 siRNA. After culturing in osteogenic medium for three days, ALP activity assay **c** or real-time PCR analyses for ALP, OSX, OCN, and COL1A1 mRNA were performed **d***–***f**. ***p < 0.001; **p < 0.01; *p < 0.05. *t*-test

SIK1 regulates CREB transcription activity by phosphorylating and thereby suppressing CRTC1 in neurons^21^ and CREB is an important transcription factor for osteogenesis^50,51^. Moreover, SIK activity has recently been implicated in regulation of osteocyte behavior in response to PTH^37^. Based on these previous reports and our current results showing the involvement of SIK1 activity in BMP2-induced osteogenesis (Figs. 1 and 2 and Supplemental Figs. 3 and 6), we hypothesized that SIK1 may modulate osteogenesis via CRTC and CREB at the downstream of BMP2. We first examined the possibility of CRTC1 as a target of SIK1 in osteogenesis. In BMP2-treated C2C12 cells, SIK1-WT overexpression greatly increased CRTC1 phosphorylation, whereas T182A overexpression suppressed CRTC1 phosphorylation (Fig. 4a). Phosphorylated CRTCs are sequestered in the cytoplasm^21,22,52^. Consistently, treatment with the SIK inhibitor HG-9-91-01 increased nuclear and decreased cytoplasmic CRTC1 levels in primary preosteoblasts (Fig. 4b). We next examined the effects of CRTC1 downregulation on the enhancement of osteogenesis by SIK1 knockdown. In preosteoblasts cotransfected with SIK1 siRNA and CRTC1 siRNA, mRNA levels of SIK1 and CRTC1 were specifically downregulated (Supplemental Fig. 8). The addition of CRTC1 siRNA blunted the increase in ALP activity by SIK1 knockdown (Fig. 4c). Consistently, the increases in mRNA levels of osteogenic marker genes by SIK1 siRNA were significantly attenuated by CRTC1 siRNA (Fig. 4d–g). These results suggest that SIK1 regulates osteoblast differentiation via CRTC1.

### SIK1 regulates osteoblast differentiation through a CREB–Id1 axis

**Fig. 5:**
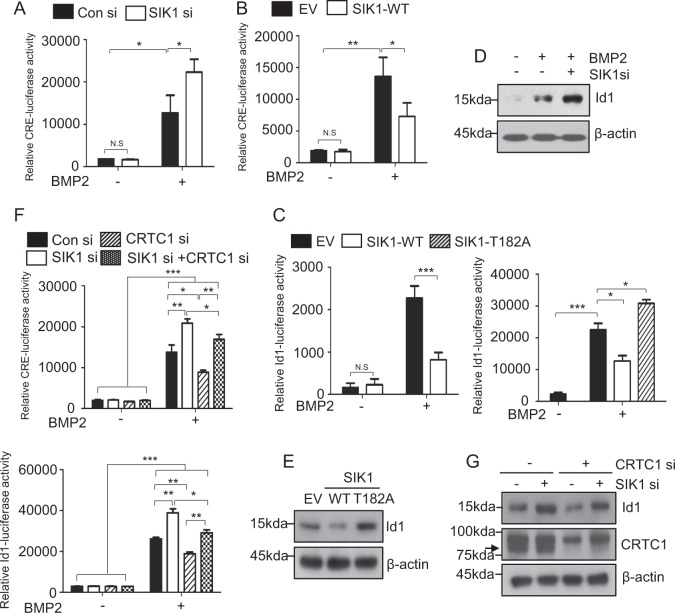
The CREB/Id1 axis is a downstream target of SIK1. **a**, **b** A CRE-luciferase reporter plasmid together with indicated siRNA or pcDNA3 plasmid were transfected to C2C12 cells. Cells were treated with vehicle or hBMP2 (150 ng/mL) for 12 h. Luciferase assay was performed with cell lysates. **c** An Id1 reporter together with pcDNA3, SIK1-WT, or SIK1-T182A plasmid were transfected to C2C12 cells. After treating with hBMP2 (150 ng/mL) for 12 h, cell lysates were subjected to luciferase assay. **d**, **e** C2C12 cells transfected with SIK1 siRNA or indicated SIK1 plasmid were treated with hBMP2 (150 ng/mL) for 18 h. The level of Id1 protein was determined by Western blotting. Relative Id1 band intensities are presented as histograms. **f** Primary preosteoblasts cotransfected with SIK1 siRNA and CRTC1 siRNA were stimulated with hBMP2 (150 ng/mL) for 12 h. CRE and Id1 luciferase assays were performed. **g** Primary preosteoblasts cotransfected with SIK1 siRNA and CRTC1 siRNA were stimulated with hBMP2 (150 ng/mL) for 18 h. Id1 protein levels were analyzed by Western blotting. The levels of Id1 and CRTC1 were normalized to β-actin. The arrow indicates the CRTC1 band below a nonspecific band. ***p < 0.001; **p < 0.01; *p < 0.05. N.S., not significant. *t*-test and one-away ANOVA

As the function of SIK1 in osteogenesis regulation was found to be mediated by the CREB coactivator CRTC1, we next examined the effects of SIK1 knockdown in the transcriptional activity of CREB by using a CRE-reporter plasmid. The activity of CRE-luciferase reporter was increased by BMP2 and was further increased by SIK1 knockdown (Fig. 5a). On the contrary, SIK1 overexpression suppressed the activity of BMP2-stimulated CRE reporter activity (Fig. 5b). CREB can bind the CRE site in the promoter of Id1, an early response gene induced by BMPs^53,54^. Therefore, we evaluated the potential role of SIK1 in BMP2-dependent Id1 regulation. Similar to previous reports^40,55^, we observed the stimulation of Id1 promoter activity by BMP2 (Fig. 5c). This stimulation was attenuated by SIK1-WT and augmented by SIK1-T182A (Fig. 5c). Consistently, the BMP2 induction of Id1 protein expression was potentiated by SIK1 siRNA (Fig. 5d) and reduced by SIK1-WT (Fig. 5e). Furthermore, the induction of both CRE and Id1 promoter activities by SIK1 knockdown was suppressed by the cotransfection of CRTC1 siRNA (Fig. 5f). The protein expression pattern of Id1 was consistent with the results of reporter assays (Fig. 5g). These data indicate that SIK1 inhibits osteoblast differentiation by interfering with the CRTC1–CREB–Id1 axis.

### SIK1 KO mice display enhanced bone formation

**Fig. 6:**
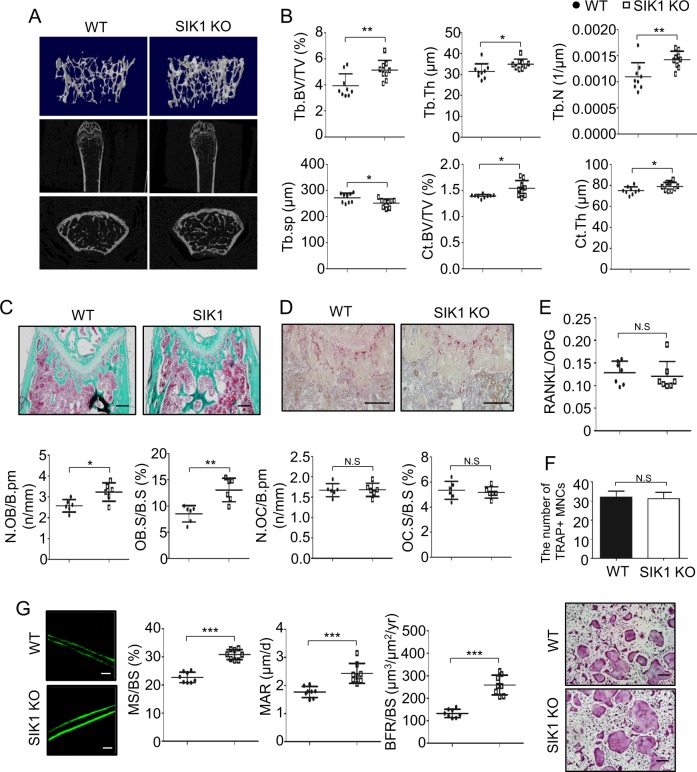
SIK1 deficiency increases bone formation in vivo. **a**, **b** Femurs of 10-week-old SIK1 KO and littermate WT female mice were analyzed by μCT (*n* = 9 per group). Three-dimensional images of trabecular bone reconstructed with Skyscan CTvol software (upper), and two-dimensional sagittal (middle) and transaxial (bottom) images produced with the DataViewer software are shown **a**. Trabecular and cortical bone parameters obtained by μCT analyses are presented. Trabecular bone parameters; trabecular bone volume/total volume (Tb.BV/TV), trabecular thickness (Tb.Th), trabecular number (Tb.N), and separation (Tb.sp), and cortical bone parameters; cortical BV/TV (Ct.BV/TV) and cortical thickness (Ct.Th) (**b**). **c**, **d** Decalcified femur sections were stained with Goldner’s trichrome **c** and for TRAP **d**. Stained sections were analyzed with Osteomeasure software to obtain indices of osteoblast number per bone perimeter (N.OB/B.pm), osteoblast surface per bone surface (OB.S/B.S), osteoclast number per bone perimeter (N.OC/B.Pm), and osteoclast surface per bone surface (OC.S/BS). **e** Serum RANKL and OPG were assessed with ELISA kits. **f** BMMs from WT or SIK1 KO mice were cultured with M-CSF (30 ng/mL) and RANKL (150 ng/mL). After four days, cells were stained for TRAP and TRAP-positive multinuclear cells were counted. **g** WT and SIK1 KO mice were injected with calcein as described in Materials and Methods. Femurs without decalcification were embedded in resin. Representative fluorescence images of sectioned slices are shown. Mineralized surface per bone surface (MS/BS), mineral apposition rate (MAR), and bone formation rate per bone surface (BFR/BS) parameters were analyzed with the Osteomeasure software. ***p < 0.001; **p < 0.01; *p < 0.05. N.S., not significant. *t*-test. Scale bars, 200 μm **c**, **d** or 20 μm **g**

To gain evidence for the role of SIK1 in vivo, we next evaluated the bone phenotype of SIK1 KO mice. When femurs of 10-week-old female KO or WT mice were analyzed by μCT, three-dimensional images showed trabecular bone mass evidently higher in KO than in WT mice (Fig. 6a). μCT analyses indicated that KO had significantly higher trabecular and cortical bone volume compared with WT (Fig. 6b). In male mice, KO showed significantly higher trabecular number and thickness values with a tendency for increased trabecular and cortical bone volume values versus WT (Supplemental Fig. 9). Histomorphometric analyses showed that the osteoblast parameters were significantly increased by SIK1 deficiency (Fig. 6c), but the osteoclast parameters were not different between WT and KO (Fig. 6d). In addition, there was also no difference regarding RANKL/OPG ratio in the sera of WT and KO mice (Fig. 6e). Osteoclastogenic cultures of BMMs from SIK1 KO and WT mice showed no difference between the two groups with respect to the number of osteoclasts identified by TRAP staining and the expression levels of osteoclast marker genes NFATc1 and TRAP (Fig. 6f and Supplemental Fig. 4D). Furthermore, calcein double labeling experiments revealed a higher bone anabolic activity in KO mice (Fig. 6g). Based on these in vivo and in vitro analyses, it is reasonable to conclude that the increased bone mass in SIK1 KO mice is mainly due to the effect of SIK1 deficiency in osteoblast-lineage cells, not to an indirect systemic attribution or an inhibition of osteoclast activity.

### SIK1 is selectively downregulated during osteogenesis via a PKA-dependent mechanism

**Fig. 7:**
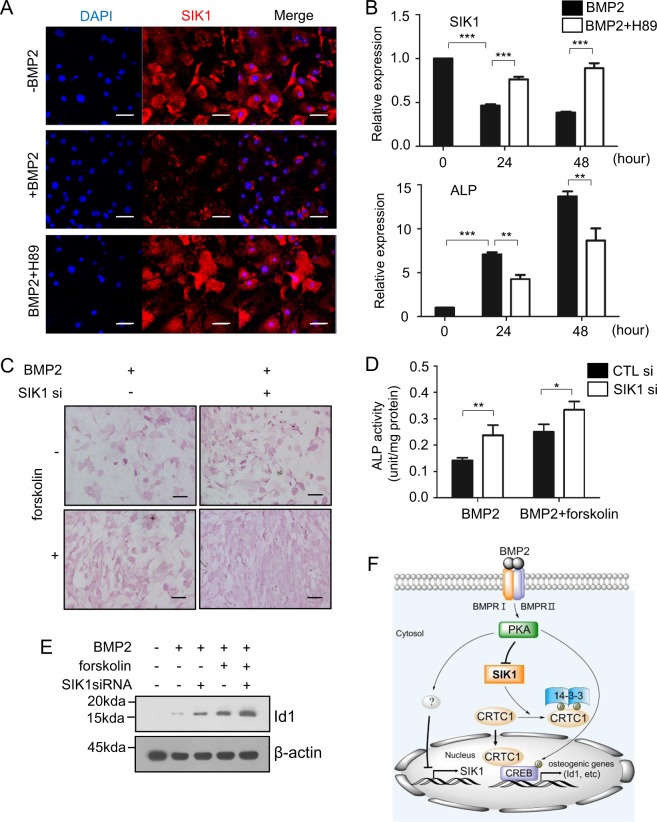
BMP2 downregulates SIK1 via PKA signaling. **a** Primary preosteoblasts were treated with BMP2 (150 ng/mL) for two days in the absence or presence of H89 (10 μM). The protein expression of SIK1 was analyzed by confocal microscopy (Red: SIK1; Blue: DAPI). **b** Primary preosteoblasts were treated with BMP2 (150 ng/mL) in the absence or presence of H89 (10 μM). The SIK1 and ALP mRNA levels were analyzed by real-time PCR. **c**, **d** Primary preosteoblasts transfected with control or SIK1 siRNA were treated with BMP2 (150 ng/mL) for three days in the absence or presence of forskolin (1 μM). Cells were stained for ALP **c** or cell lysates were subjected to ALP activity assay **d**. **e** C2C12 cells were transfected with SIK1 siRNA and stimulated with hBMP2 (150 ng/mL) in the absence or presence of forskolin (10 μM) for 18 h. Id1 protein expression was analyzed by Western blotting, and the level of Id1 was normalized to that of β-actin. ***p < 0.001; **p < 0.01, *p < 0.05. Scale bars, 50 μm **a** or 200 μm **c**. **f** A schematic illustration for the role of SIK1 during osteogenesis. In the absence of BMP2 signaling, SIK1 phosphorylates CRTC1 and thereby inhibits its nuclear translocation, leading to a suppression of CREB target genes. In response to BMP2, SIK1 level is downregulated and SIK1 activity is inhibited by PKA-dependent mechanisms. Under this SIK1-repressed condition, dephosphorylated CRTC1 translocates into the nucleus and stimulates CREB activity for induction of osteogenic genes including Id1

As SIK1 activity negatively regulated bone formation and the expression of SIK1 decreased in the osteogenic medium containing β-glycerophosphate and ascorbic acid, we next examined whether BMP2 would affect SIK1 expression. BMP2 treatment decreased the expression of SIK1 protein as well as mRNA (Fig. 7a, b). Intriguingly, the BMP2-induced downregulation of SIK1 was attenuated by H89, a PKA inhibitor (Fig. 7a, b). In line with these observations, the PKA activator forskolin enhanced BMP2-induced osteoblast differentiation, while SIK1 knockdown further potentiated the effect of forskolin (Fig. 7c, d). Forskolin-dependent CRE and Id1 promoter activities were also attenuated by SIK1-WT, but not by SIK1-T182A (Supplemental Fig. 10). In addition, forskolin augmented the BMP2 upregulation of Id1 protein level, which was further increased by SIK1 siRNA (Fig. 7e). These results suggest that the PKA-mediated downregulation of SIK1 is essential for osteogenic differentiation, as SIK1 inhibits the CREB activity involved in the transcription of multiple osteogenic genes.

## Discussion

In this study, we revealed that SIK1 plays an important role in bone metabolism by regulating osteogenesis in vitro and in vivo. Based on our findings, we propose that the suppression of SIK1 activity and subsequent activation of the CRTC1–CREB pathway is a crucial event in bone anabolism.

SIKs have been previously implicated in multiple processes^39,56–60^. For skeletal physiology, with the exception of SIK1, other members SIK2 and SIK3 have recently been implicated. These include that SIK3 KO mice revealed a severe inhibition of chondrocyte hypertrophy^38^. Moreover, SIK2 was shown to mediate bone-forming action of PTH in osteocytes^37^. In this study, PTH induced the repression of *Sost*, a gene encoding the Wnt inhibitor sclerostin, by stimulating the inactivation of SIK2 in Ocy454 osteocytic cells. Lowered SIK2 activity results in the underphosphorylation of HDAC4 and HDAC5^23,24^, causing translocation to the nucleus for repression of Mef2c involved in *Sost* transcription^37^. Consistent with bone anabolic effects of intermittent PTH, treatment with pan-SIK inhibitors increased bone formation^37^. Whether SIK1 could also function in the osteocyte response to PTH is not clear. However, the findings of that PTH- and SIK-inhibitor-induced *Sost* suppression did not occur in SIK2-knockout and SIK2/3-knockdown osteocytes, respectively^37^, indicate no significant part played by SIK1 in osteocytes and point to the specific roles of each SIK member in the control of osteoblast-lineage cells in responding to different signals. Nevertheless, both our results showing the role of SIK1 in osteoblasts and the previous report revealing the role of SIK2 in osteocytes support the clinical value of SIK inhibitors as bone anabolic agents.

PKA is one of the major molecules leading to the stimulation of CREB, a crucial osteogenic factor^51,61^. In response to elevate cAMP levels, activated PKA directly phosphorylates CREB to enhance its transcription activity^62,63^. Recent findings add another layer of CREB stimulation by PKA, achieved through SIKs^22,28^. In this mechanism, the PKA-dependent phosphorylation of SIKs inhibits phosphorylating CRTCs, unleashing CRTCs from cytoplasmic retention and facilitating nuclear translocation for CREB binding^28^. This PKA inhibition of the catalytic activity of SIKs may be reinforced by a regulation of SIKs expression levels. Indeed, we observed a decrease in SIK1 levels via forskolin treatment, a PKA activator (data not shown), or under osteogenic medium (Supplemental Fig. 1). In addition, H89, a PKA inhibitor, blocked the BMP2-dependent reduction of SIK1 levels (Fig. 7a). Therefore, it appears that SIK1 suppression is a requisite in driving osteogenic differentiation, which is ensured by the dual actions of PKA in reducing SIK1 expression level as well as SIK1 catalytic activity (Fig. 7f). Interestingly, SIK1 abundance was suggested to be critical for myogenesis and SIK1 mRNA and protein levels were shown to increase during myoblast differentiation^59^. Thus, our results clearly point to contrasting directions in the regulation of expression level of SIK1 during osteogenesis and myogenesis. Whether this distinctive expression pattern of SIK1 is a key element in the fate determination of mesenchymal progenitor cells of osteoblasts and myoblasts is an intriguing question to be explored in further work.

Recently, SIK has been implicated in RANKL-induced osteoclastogenesis^64^. In the study, HG-9-91-01 suppressed osteoclast differentiation. Either SIK2- or SIK3-silencing by CRISPR/Cas9 system in RAW264.7 reduced osteoclastogenesis, suggesting that SIK2 and SIK3 may play nonredundant roles, with the lack of SIK2 not being compensated for by the presence of SIK3 and vice versa. Although whether SIK1 was also involved in osteoclastogenesis could not be addressed in the report, our observations indicated inconsistent effects of SIK1 siRNA in BMMs (data not shown). We also observed no differences in osteoclast number between WT and SIK1 KO bones and in the RANKL/OPG ratio between WT and KO mice sera (Fig. 6). Although the answer to whether SIK1 is dominant in osteoblast regulation while SIK2/3 are more important in osteoclast regulation remains elusive, the demonstration of a predominant role of SIK2 in PTH responses in osteocytes^37^ suggests a specificity in the actions of SIK each member. However, our data showing the control of osteoblast differentiation by SIK1, the study on SIK2 function for PTH responses in osteocytes^37^, and the demonstration of SIK2/3 involvement in osteoclastogenesis^64^ all indicate that SIK inhibition is beneficial for conditions requiring bone mass increases, such as osteoporosis.

We found that the major target of SIK1 in regulating osteogenesis is CRTC1. CRTCs enhance CREB transcription activity by binding to the basic leucine zipper domain of CREB in the nucleus^52,65^. CRTCs in phosphorylated states are sequestered in the cytoplasm by 14-3-3 binding and thus dephosphorylation is required for the nuclear translocation of CRTCs^21,22,65^. To date, studies probing the critical role of CRTCs in osteogenesis has been scanty. In this study, we revealed that the reduction in SIK1 activity during osteoblast differentiation led to a decrease in CRTC1 phosphorylation and an increase in nuclear CRTC1 levels. More importantly, our results showed that CRTC1 knockdown almost completely blocked the increasing effects of SIK1 knockdown on osteoblast differentiation (Fig. 4), suggesting CRTC1, among three CRTCs, to be the major player in the control of osteogenesis. Enhanced CREB activity may be linked to Id gene expression as a CREB binding site is present in the Id1 gene promoter^66^ and CREB-stimulators like dibutyryl-cAMP and forskolin increased Id transcription^30,67,68^. In addition, BMP2 increases Id levels in osteoblast-lineage cells and myoblasts (C2C12)^55,69,70^ and Ids have positive roles for bone formation in mice^54^. In our study, BMP2-induction of Id1 was regulated by the SIK1– CRTC1 pathway and SIK KO cells had a higher level of Id1 expression versus WT. Therefore, the CRTC1–CREB–Id1 pathway appears to be primarily responsible for the osteogenesis regulation by SIK1.

Ids function as negative regulators of bHLH-containing transcriptions factors by direct binding and subsequent inhibition of DNA binding of bHLH factors^71,72^. Alternatively, Id1 was shown to induce the degradation of Twist1, a bHLH factor that suppresses the activity of osteogenic factor like Runx2^73^, to increase BMP signaling^74^. As Ids counteract Twists, induction of Id1 may also be essential to the maintenance of bone homeostasis by supporting osteogenesis during bone remodeling in adults. In this study, we for the first time revealed a link between SIK1 activity regulation and BMP2-induction of Id1 for osteogenic process.

In conclusion, our results demonstrate that the suppression of SIK1 activity is critical in the initiation and progression of osteogenesis to stimulate precursor proliferation and to induce expression of osteoblastic genes. We also showed that, to achieve SIK1 inhibition, BMP2 reduces SIK1 gene expression and also catalytic activity, both in a PKA-dependent manner. The reduction in SIK1 activity leads to CRTC1–CREB activation, which turns on the osteogenesis program by stimulating the transcription of Id1 and other osteogenic genes. Recently, due to revealing side effects of anti-resorptive drugs^75,76^, developments of osteoblast-targeted anabolic agents have become of great interest^2,3^. Therefore, SIK1 inhibition is worth as a bone anabolic strategy for the treatment pathogenic conditions like osteoporosis and bone fracture.

## Supplementary information

supplemenetal Figures and Tables

## References

[1] Chen JS (2011). Antiresorptive therapies for osteoporosis: a clinical overview. Nat. Rev. Endocrinol..

[2] Corrado A (2017). Osteoblast as a target of anti-osteoporotic treatment. J. Postgrad. Med.

[3] Augustine M (2013). Parathyroid hormone and parathyroid hormone-related protein analogs as therapies for osteoporosis. Curr. Osteoporos. Rep..

[4] Lotinun S (2002). Differential effects of intermittent and continuous administration of parathyroid hormone on bone histomorphometry and gene expression. Endocrine.

[5] Meyer, L. FDA approves new treatment for osteoporosis in postmenopausal women at high risk of fracture. www.fda.gov (12 April 2019).

[6] Long F (2011). Building strong bones: molecular regulation of the osteoblast lineage. Nat. Rev. Mol. Cell Biol..

[7] Rutkovskiy A (2016). Osteoblast Differentiation at a Glance. Med. Sci. Monit. Basic Res..

[8] Wu M (2016). TGF-β and BMP signaling in osteoblast, skeletal development, and bone formation, homeostasis and disease. Bone Res..

[9] Miyazono K (2005). BMP receptor signaling: transcriptional targets, regulation of signals, and signaling cross-talk. Cytokine Growth Factor Rev..

[10] Greenblatt MB (2010). Thep38 MAPK pathway is essential for skeletogenesis and bone homeostasis in mice. J. Clin. Invest..

[11] Ge CX (2012). Interactions between extracellular signal-regulated kinase 1/2 and P38 Map kinase pathways in the control of RUNX2 phosphorylation and transcriptional activity. J. Bone Min. Res..

[12] Lee KS (2002). Both the Smad and p38 MAPK pathways play a crucial role in Runx2 expression following induction by transforming growth factor-beta and bone morphogenetic protein. Oncogene.

[13] Huang W (2007). Signaling and transcriptional regulation in osteoblast commitment and differentiation. Front Biosci..

[14] Hardie DG (1997). The AMP-activated protein kinase-fuel gauge of the mammalian cell?. Eur. J. Biochem..

[15] Wein MN (2018). Salt-inducible kinases: physiology, regulation by cAMP, and therapeutic potential. Trends Endocrin. Met..

[16] Lizcano JM (2004). LKB1 is a master kinase that activates 13 kinases of the AMPK subfamily, including MARK/PAR‐1. EMBO J..

[17] Jaleel M (2005). Identification of the sucrose non-fermenting related kinase SNRK, as a novel LKB1 substrate. FEBS Lett..

[18] Henriksson E (2012). The AMPK-related kinase SIK2 is regulated by cAMP via phosphorylation at Ser(358) in adipocytes. Biochem. J..

[19] Takemori H (2002). ACTH-induced nucleocytoplasmic translocation of salt-inducible kinase: Impication in the protein kinase A-activated gene transcription in mouse adrenocortical tumor cells. J. Biol. Chem..

[20] Takemori H (2007). TORC‐SIK cascade regulates CREB activity through the basic leucine zipper domain. FEBS J..

[21] Li S (2009). TORC1 regulates activity-dependent CREB-target gene transcription and dendritic growth of developing cortical neurons. J. Neurosci..

[22] Katoh Y (2006). Silencing the constitutive active transcription factor CREB by the LKB1-SIK signaling cascade. FEBS J..

[23] Berdeaux R (2007). SIK1 is a class II HDAC kinase that promotes survival of skeletal myocytes. Nat. Med.

[24] van der Linden AM (2007). KIN‐29 SIK regulates chemoreceptor gene expression via an MEF2 transcription factor and a class II HDAC. EMBO J..

[25] Yong Kim S (2013). Salt-Inducible Kinases 1 and 3 negatively regulate toll-like receptor 4-mediated signal. Mol. Endocrinol..

[26] Sanosaka M (2015). Salt-inducible kinase 3 deficiency exacerbates lipopolysaccharide-induced endotoxin shock accompanied by increased levels of pro-inflammatory molecules in mice. Immunology.

[27] Lombardi MS (2016). SIK inhibition in human myeloid cells modulates TLR and IL-1R signaling and induces an anti-inflammatory phenotype. J. Leukoc. Biol..

[28] Sonntag T (2018). 14-3-3 proteins mediate inhibitory effects of cAMP on salt-inducible kinases (SIKs). FEBS J..

[29] Viale-Bouroncle S (2015). A protein kinase A (PKA)/beta-catenin pathway sustains the BMP2/DLX3-induced osteogenic differentiation in dental follicle cells (DFCs). Cell Signal..

[30] Zhang H (2015). Activation of PKA/CREB signaling is involved in BMP9-induced osteogenic differentiation of mesenchymal stem cells. Cell Physiol. Biochem..

[31] Zhao L (2006). Downregulation of cAMP-dependent protein kinase inhibitor gamma is required for BMP-2-induced osteoblastic differentiation. Int J. Biochem. Cell Biol..

[32] Datta NS (2009). PTH and PTHrP signaling in osteoblasts. Cell Signal.

[33] Yang R (1996). Signal transduction pathways mediating parathyroid hormone stimulation of bone sialoprotein gene expression in osteoblasts. J. Biol. Chem..

[34] Jongen JW (1993). Parathyroid hormone-induced changes in alkaline phosphatase expression in fetal calvarial osteoblasts: differences between rat and mouse. J. Cell Physiol..

[35] Kondo H (1997). Temporal changes of mRNA expression of matrix proteins and parathyroid hormone and parathyroid hormone-related protein (PTH/PTHrP) receptor in bone development. J. Bone Min. Res..

[36] Vilardaga J-P (2011). Molecular basis of parathyroid hormone receptor signaling and trafficking: a family B GPCR paradigm. Cell Mol. Life Sci..

[37] Wein MN (2016). SIKs control osteocyte responses to parathyroid hormone. Nat. Commun..

[38] Sasagawa S (2012). SIK3 is essential for chondrocyte hypertrophy during skeletal development in mice. Development.

[39] Kim MJ (2015). Salt-Inducible Kinase 1 terminates cAMP signaling by an evolutionarily conserved negative-feedback loop in beta-cells. Diabetes.

[40] Korchynskyi O (2002). Identification and functional characterization of distinct critically important bone morphogenetic protein-specific response elements in the Id1 promoter. J. Biol. Chem..

[41] Kim H (2017). Extracellular S100A4 negatively regulates osteoblast function by activating the NF-kappaB pathway. BMB Rep..

[42] Chen J (2015). mTORC1 signaling promotes osteoblast differentiation from preosteoblasts. PLoS ONE.

[43] Traianedes K (1998). 5-Lipoxygenase metabolites inhibit bone formation in vitro. Endocrinology.

[44] Tare RS (2002). Pleiotrophin/osteoblast-stimulating factor 1: dissecting its diverse functions in bone formation. J. Bone Min. Res..

[45] Katagiri T (1990). The non-osteogenic mouse pluripotent cell line, C3H10T1/2, is induced to differentiate into osteoblastic cells by recombinant human bone morphogenetic protein-2. Biochem. Biophys. Res. Commun..

[46] Kim HJ (2012). Plasma membrane calcium ATPase regulates bone mass by fine-tuning osteoclast differentiation and survival. J. Cell Biol..

[47] Yoon SH (2011). Adenylate cyclase and calmodulin-dependent kinase have opposite effects on osteoclastogenesis by regulating the PKA-NFATc1 pathway. J. Bone Min. Res..

[48] Oh JE (2012). PlexinA2 mediates osteoblast differentiation via regulation of Runx2. J. Bone Min. Res..

[49] Chang EJ (2008). Brain-type creatine kinase has a crucial role in osteoclast-mediated bone resorption. Nat. Med.

[50] Kim JM (2013). An activator of the cAMP/PKA/CREB pathway promotes osteogenesis from human mesenchymal stem cells. J. Cell Physiol..

[51] Siddappa R (2008). cAMP/PKA pathway activation in human mesenchymal stem cells in vitro results in robust bone formation in vivo. Proc. Natl Acad. Sci. USA.

[52] Screaton RA (2004). The CREB Coactivator TORC2 Functions as a Calcium- and cAMP-Sensitive Coincidence Detector. Cell.

[53] Ohta Y (2008). Cyclic AMP enhances Smad-mediated BMP signaling through PKA-CREB pathway. J. Bone Min. Metab..

[54] Maeda Y (2004). Inhibitory helix-loop-helix transcription factors Id1/Id3 promote bone formation in vivo. J. Cell Biochem..

[55] Ogata T (1993). Bone morphogenetic protein 2 transiently enhances expression of a gene, Id (inhibitor of differentiation), encoding a helix-loop-helix molecule in osteoblast-like cells. Proc. Natl Acad. Sci. USA.

[56] Takemori H (2003). Salt-inducible kinase-mediated regulation of steroidogenesis at the early stage of ACTH-stimulation. J. Steroid Biochem Mol. Biol..

[57] Okamoto M (2004). Salt-inducible kinase in steroidogenesis and adipogenesis. Trends Endocrinol. Metab..

[58] Taub M (2010). Targeting of renal proximal tubule Na,K-ATPase by salt-inducible kinase. Biochem. Biophys. Res. Commun..

[59] Stewart R (2013). Regulation of SIK1 abundance and stability is critical for myogenesis. Proc. Natl Acad. Sci. USA.

[60] Gallo EF (2011). Balancing Life and Death in the Ischemic Brain: SIK and TORC Weigh. Neuron.

[61] Zhao M (2009). CREB induces BMP2 transcription in osteoblasts and CREB knockout reduces bone mass in mice. Bone.

[62] Gonzalez GA (1989). Cyclic AMP stimulates somatostatin gene transcription by phosphorylation of CREB at serine 133. Cell.

[63] Lin RZ (1998). Phosphorylation of the cAMP response element-binding protein and activation of transcription by alpha1 adrenergic receptors. J. Biol. Chem..

[64] Lombardi MS (2017). Salt-inducible kinases (SIK) inhibition reduces RANKL-induced osteoclastogenesis. PLoS ONE.

[65] Conkright MD (2003). TORCs: transducers of regulated CREB activity. Mol. Cell.

[66] Lopez-Rovira T (2002). Direct binding of Smad1 and Smad4 to two distinct motifs mediates bone morphogenetic protein-specific transcriptional activation of Id1 gene. J. Biol. Chem..

[67] Doorn J (2012). Forskolin enhances in vivo bone formation by human mesenchymal stromal cells. Tissue Eng. Part A.

[68] Scobey MJ (2004). The Id2 transcriptional repressor is induced by follicle-stimulating hormone and cAMP. J. Biol. Chem..

[69] Peng Y (2004). Inhibitor of DNA binding/differentiation helix-loop-helix proteins mediate bone morphogenetic protein-induced osteoblast differentiation of mesenchymal stem cells. J. Cell Biochem..

[70] Katagiri T (1994). Bone morphogenetic protein-2 converts the differentiation pathway of C2C12 myoblasts into the osteoblast lineage. J. Cell Biol..

[71] Benezra R (1990). The protein Id: A negative regulator of helix-loop-helix DNA binding proteins. Cell.

[72] Miyazono K (2002). Id: a target of BMP signaling. Sci. STKE.

[73] Bialek P (2004). A twist code determines the onset of osteoblast differentiation. Dev. Cell.

[74] Hayashi M (2007). Comparative roles of Twist-1 and Id1 in transcriptional regulation by BMP signaling. J. Cell Sci..

[75] Skjødt MK (2019). Side effects of drugs for osteoporosis and metastatic bone disease. Br. J. Clin. Pharm..

[76] Kennel KA (2009). Adverse effects of bisphosphonates: implications for osteoporosis management. Mayo Clin. Proc..

