# Supplementary material for: Salt-inducible kinase 1 regulates bone anabolism via the CRTC1–CREB–Id1 axis
Source: Cell Death Dis. 2019 Oct 31;10(11):826. doi: 10.1038/s41419-019-1915-4 (PMC6823377; doi:10.1038/s41419-019-1915-4)
Supplement: Supplementary file 1 — supplemenetal Figures and Tables [file 41419_2019_1915_MOESM1_ESM.pdf]

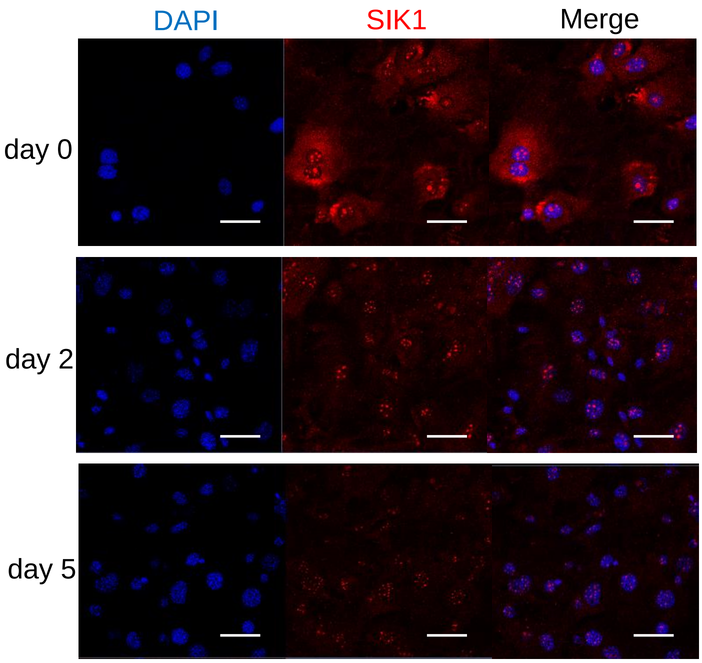

Supplemental Figure 1. SIK1 was downregulated during osteoblast differentiation. Primary preosteoblasts were cultured in osteogenic medium containing β-glycerophosphate and ascorbic acid for five days. Cells at Days 0, 2, and 5 were incubated with anti-SIK1 followed by Cy3-conjugated secondary antibody. Cells mounted with a mounting solution containing DAPI were observed under a confocal microscope.

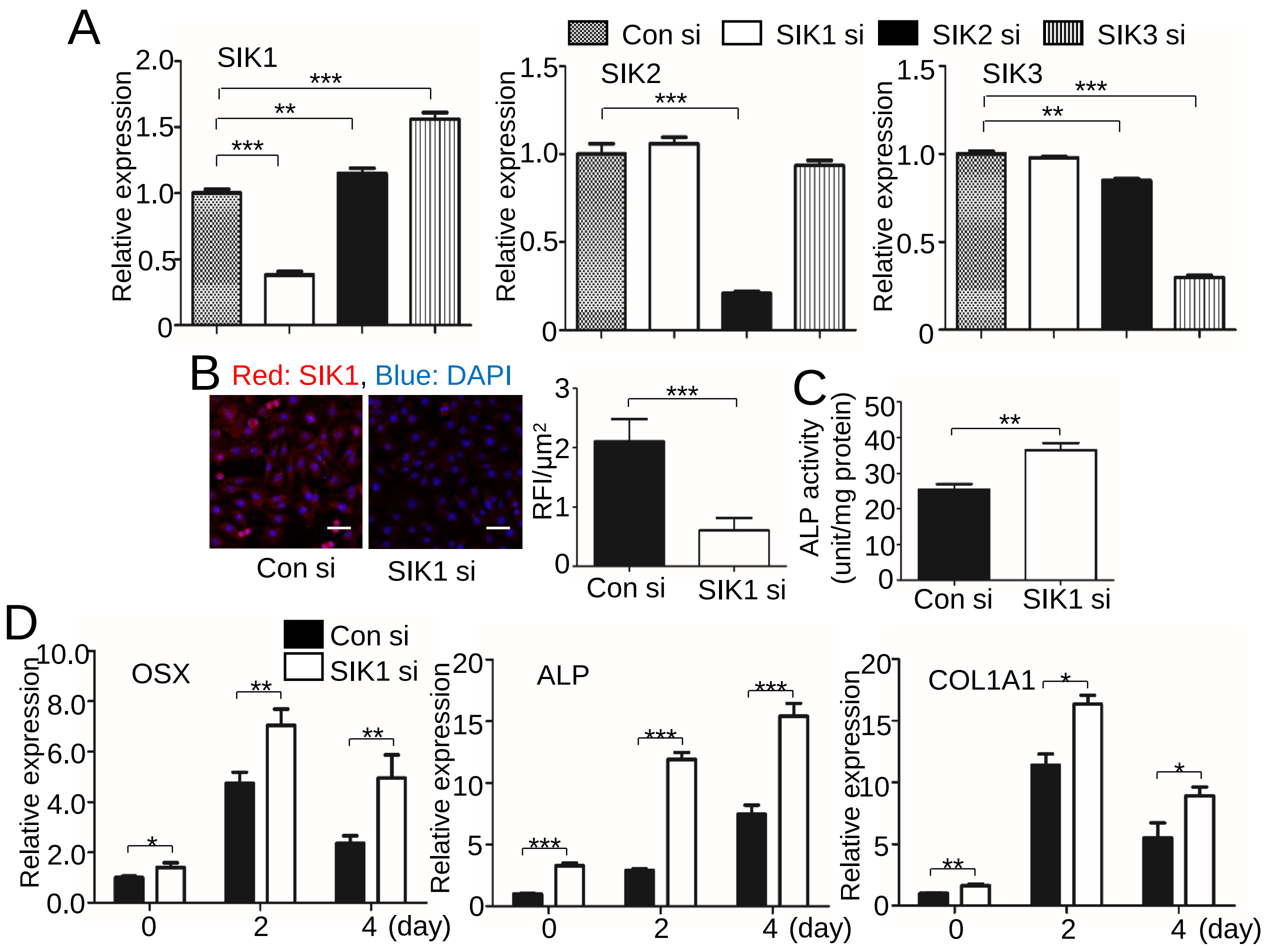

Supplemental Figure 2. Specific gene knockdown of SIK1 enhances osteogenesis in mouse preosteoblasts. (A-E) Primary preosteoblasts were transfected with siRNA targeted to each SIK gene or control siRNA, then cultured with osteogenic medium. The mRNA levels of SIK1, SIK2, and SIK3 were determined by real-time PCR *(A)*. To quantify the extent of SIK1 knockdown at the protein level, cells were subjected to immunofluorescence microscopy. The fluorescence intensity of Cy3-labeled cells was analyzed by confocal microscopy *(B)*. Cells were lysed for quantitative liquid ALP assay at Day 3 *(C)*. The mRNA expression levels of OSX, ALP, and COL1A1 were also analyzed by real-time PCR *(D)* ***, $p < 0.001$; **, $p < 0.01$; *, $p < 0.05$ versus control. *t*-test.

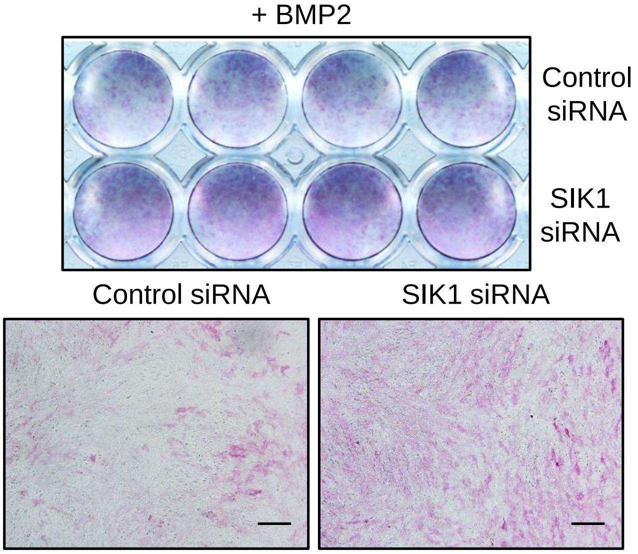

Supplemental Figure 3. SIK1 knockdown increases BMP2-induced osteoblastic differentiation of C2C12 cells. C2C12 cells were transfected with SIK1 siRNA or control siRNA. Cells were then cultured in the presence of hBMP2 (150 ng/mL) for three days. Cells were stained for ALP activity.

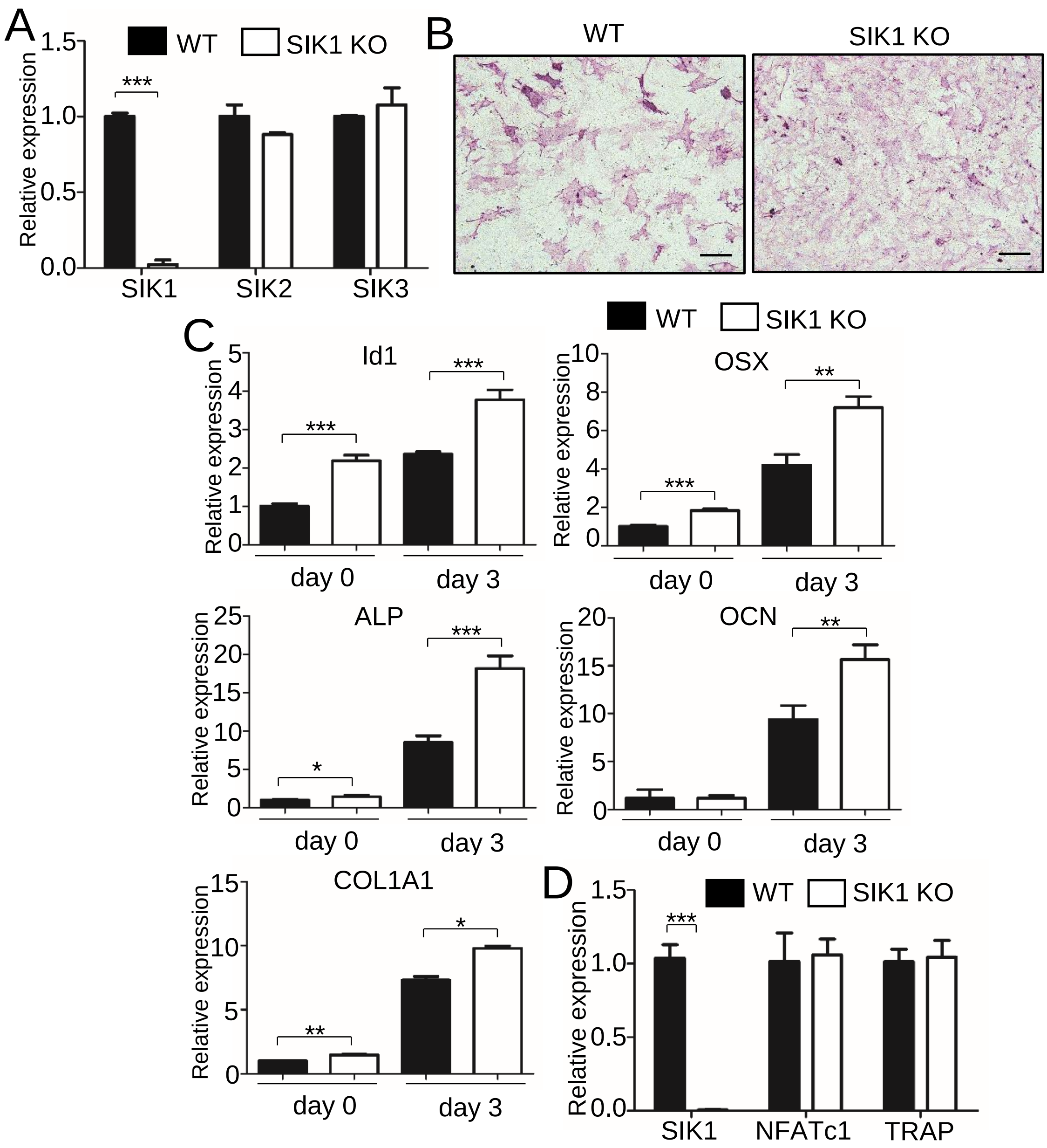

Supplemental Figure 4. SIK1 KO cells have higher osteoblastogenic activity *in vitro*. (A-C) Calvarial preosteoblasts from WT and SIK1 KO mice were cultured. The relative mRNA levels of SIK1, SIK2, and SIK3 were analyzed by real-time PCR *(A)*. Cells were cultured in the presence of osteogenic medium, then differentiated cells were subjected to ALP assays (Day 3) *(B)*. The mRNA expression levels of Id1, OSX, OCN, ALP, and COL1A1 at the indicated days were determined by real-time PCR *(C)*. (D) BMMs from WT or SIK1 KO mice were cultured with M-CSF (30 ng/mL) and RANKL (150 ng/mL). The mRNA levels of SIK1, NFATc1, and TRAP were analyzed by real-time PCR. ***, $p < 0.001$; **, $p < 0.01$; *, $p < 0.05$ versus control. *t*-test. Scale bars, 200 μm.

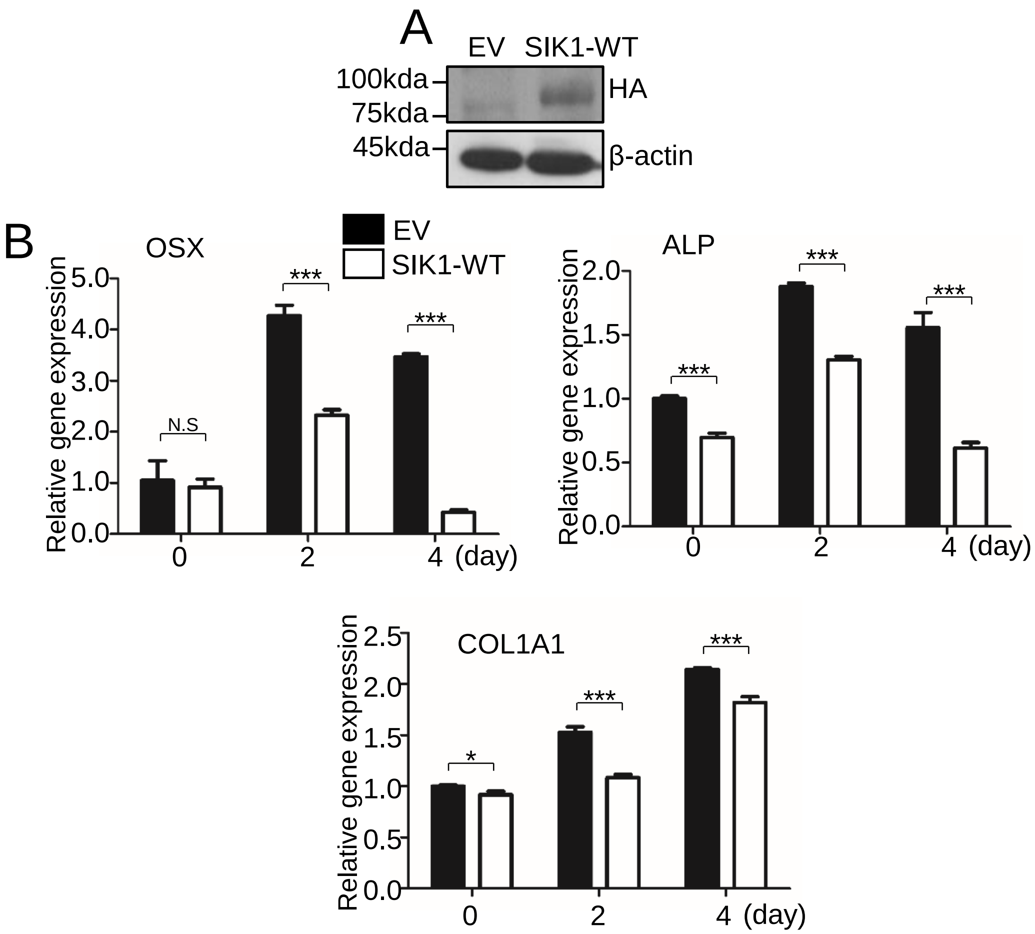

Supplemental Figure 5. SIK1 overexpression inhibits osteogenesis from clavarial preosteoblasts. (A-B) Primary preosteoblasts were transfected with pcDNA3-SIK1-HA or pcDNA3 (EV) The protein expression levels of SIK1 were analyzed by Western blotting *(A)*. Transfected cells were cultured in osteogenic medium and harvested for real-time PCR analyses for OSX and ALP mRNA levels *(B)*. ***, $p < 0.001$; *, $p < 0.05$ versus control. N.S, not significant. *t*-test.

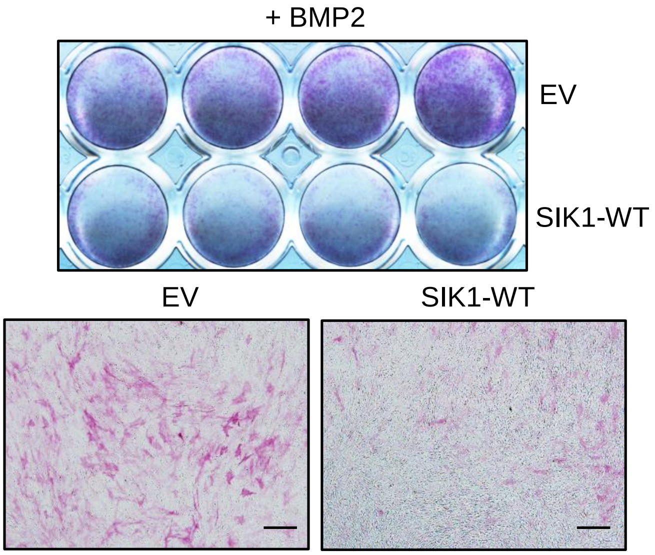

Supplemental Figure 6. SIK1 overexpression decreases BMP2-induced osteoblastic differentiation of C2C12 cells. C2C12 cells were transfected with pcDNA3-SIK1-HA or pcDNA3 (EV) plasmid. Cells were cultured with hBMP2 (150 ng/mL) for three days and then stained for ALP activity. Scale bars, 200 μm.

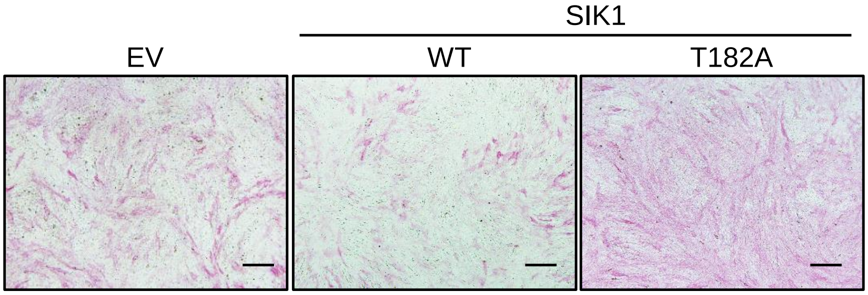

Supplemental Figure 7. SIK1 kinase activity is required to control osteoblastic differentiation of C2C12 cells. C2C12 cells were transfected with pcDNA3-SIK1-WT, pcDNA3-SIK1-T182A, or pcDNA3 (EV) plasmid. Cells were treated with hBMP2 (150 ng/mL) for three days and stained for ALP activity. Scale bars, 200μm.

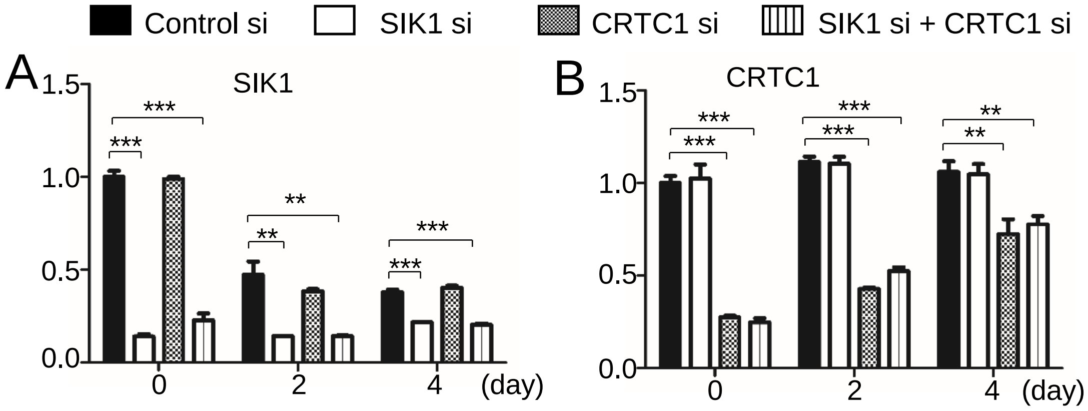

Supplemental Figure 8. Reduction of SIK1 and CRTC1 gene expression by siRNA was verified in primary preosteoblasts. (A, B) Primary preosteoblasts were transfected with SIK1 siRNA, CRTC1 siRNA, a combination of SIK1 siRNA and CRTC1 siRNA, or control siRNA. The total amount of siRNA was ensured to be the same in all groups by using additional amounts of control siRNA. Cells were cultured in osteogenic medium for four days and the mRNA levels of SIK1 *(A)* and CRTC1 *(B)* were analyzed by real-time PCR. ***, $p < 0.001$; **, $p < 0.01$ versus control. *t*-test.

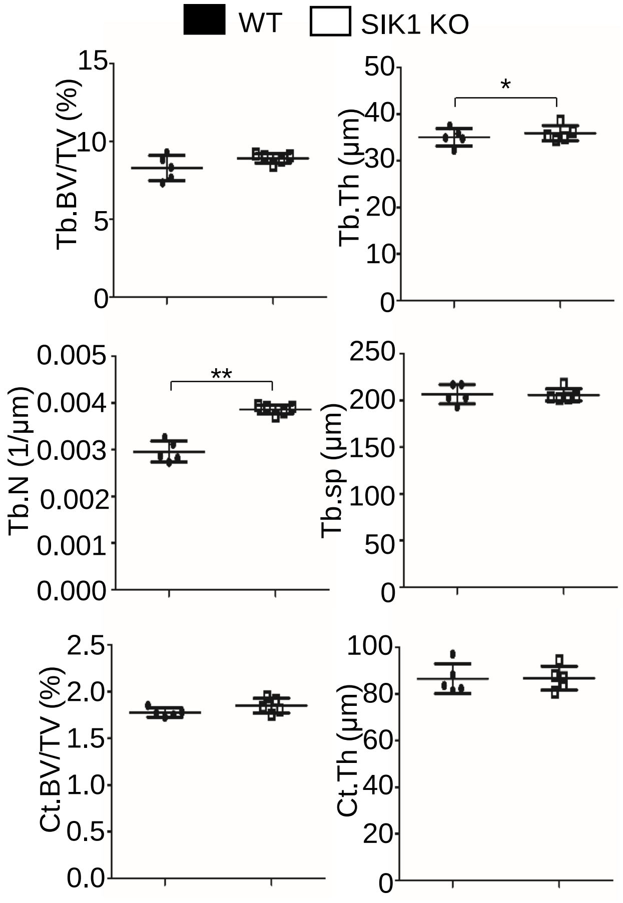

Supplemental Figure 9. Micro-CT analysis of male SIK1 KO mice. Femurs of 10-week-old SIK1 KO and littermate WT male mice were analyzed by μCT (n = 5 per group). Trabecular bone volume (Tb.BV/TV), thickness (Tb.Th), number (Tb.N), separation (Tb.sp), and cortical bone volume (Ct.BV/TV) and thickness (Ct.Th) indices were presented. **, $p < 0.01$; *, $p < 0.05$ versus WT. *t*-test.

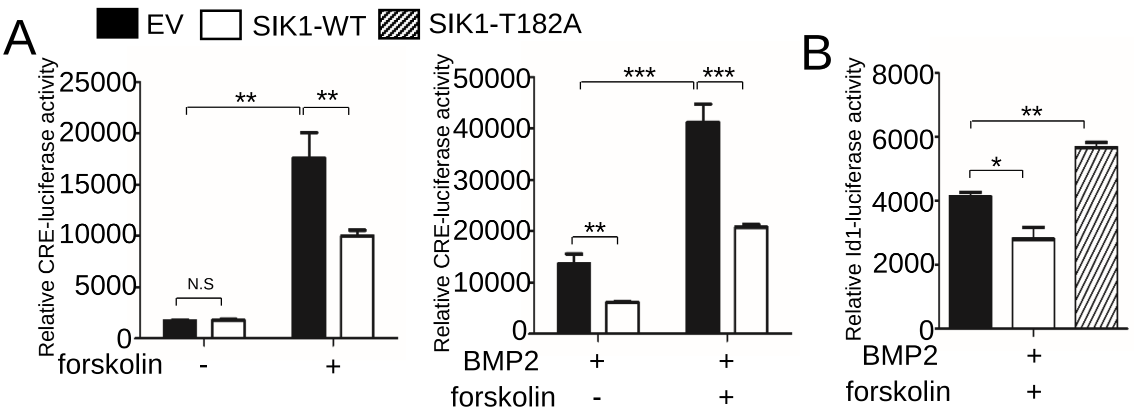

Supplemental Figure 10. SIK1 suppressed forskolin-induced CRE and Id1 promoter activities. C2C12 cells were transfected with either CRE *(A)* or Id1 promoter *(B)* luciferase reporter plasmid together with pcDNA3-SIK1-WT, pcDNA3-SIK1-T182A, or pcDNA3. Cells were stimulated with forskolin (10 μM) alone or together with hBMP2 (150 ng/mL). Cell lysates were prepared and luciferase assays were performed. ***, p < 0.001; **, p < 0.01; *, p < 0.05 versus control. *t*-test.

genes	oligonucleotides sequences
mSIK1	5'-CCA CAG CUC ACU UCA GCC CUU AUU
	3'-UAA UAA GGG CUG AAG UGA GCU GUG
mSIK2	5'-AGA AGC AGU CUC AGC UGC AAG CAU
	3'-UAU GCU UGC AGC UGA GAC UGC UUC
mSIK3	5'-CCA CAU GCU GGU GUU AGA UCC AAA
	3'-AUU UGG AUC UAA CAC CAG CAU GUG
mCRTC1	5'-GGA GAG UCA CCA CCG AGC CUC UCU
	3'-UAG AGA GGC UCG GUG GUG ACU CUC

Supplemental Table 1. Sequences of siRNA oligonucleotides

genes		primer sequences
mHPRT	F	CCT AAG ATG AGC GCA AGT TGA A
	R	CCA CAG GGA CTA GAA CAC CTG CTA A
mSIK1	F	AGA AAT TCT CCC GTG TGA CC
	R	CCA CTG CAG TTG GTA TCC AG
mSIK2	F	TCC AAG ACC TTT CGA GCA GT
	R	GGA AGA GTC GCT TCT GTT GG
mSIK3	F	TGC TGG GAA CTG TGA GTC AG
	R	TGT TCT GGA TCG TGT GGT GT
mCRTC1	F	CAG GCA GGC CAA TGC TCT GT
	R	CGC TCA GAC CCA TCA TGG CA
mALP	F	GAC TGG TAC TCG GAT AAC GA
	R	TGC GGT TCC AGA CAT AGT GG
mOSX	F	TCT ACC TGC GAC TGC CCC AA
	R	ATG CGA AGC CTT GCC GTA CA
mOCN	F	CCG GGA GCA GTG TGA GCT TA
	R	TAG ATG CGT TTG TAG GCG GTC
mId1	F	TCC TGC AGC ATG TAA TCG AC
	R	GTG GTC CCG ACT TCA GAC TC
mRANKL	F	TGG AAG GCT CAT GGT TGG AT
	R	CAT TGA TGG TGA GGT GTG CAA
mOPG	F	GCA GCC CAG TGT GCA AGG AA
	R	TTC GAT CTC CAG GTA ACG CCC
mNFATc1	F	CCA GTA TAC CAG CTC TGC CA
	R	GTG GGA AGT CAG AAG TGG GT
mTRAP	F	CGA CCA TTG TTA GCC ACA TAC G
	R	TCG TCC TGA AGA TAC TGC AGG TT
mCOL1A1	F	GCA TGG CCA AGA AGA CAT CC
	R	CCT CGG GTT TCC ACG TCT

Supplemental Table 2. Primer sequences for real-time PCR
